# First Record of* Anisakis simplex* Third-Stage Larvae (Nematoda, Anisakidae) in European Hake* Merluccius merluccius lessepsianus* in Egyptian Water

**DOI:** 10.1155/2016/9609752

**Published:** 2016-03-03

**Authors:** Yasmin Abou-Rahma, Rewaida Abdel-Gaber, Amira Kamal Ahmed

**Affiliations:** Zoology Department, Faculty of Science, Cairo University, Cairo 12613, Egypt

## Abstract

The prevalence of infection and the identification of anisakid larvae in European hake* Merluccius merluccius lessepsianus* from Hurghada City, Red Sea Governorate, Egypt, were investigated. Fish samples were collected during the period of February and November 2014. Twenty-two (36.66%) out of sixty examined fish specimens were found to be naturally infected with* Anisakis* type I larvae mostly found as encapsulated larvae in visceral organs. There was a positive relationship between host length/weight and prevalence of infection. Based on morphological, morphometric, and molecular analyses, these nematodes were identified as third-stage larvae of* Anisakis simplex*. The present study was considered as the first report of anisakid larvae from European hake in the Egyptian water.

## 1. Introduction

Nematode larvae of the genus* Anisakis* Dujardin [1] (Nematoda: Anisakidae) are common parasites of marine fish with a worldwide distribution [2]. These nematodes have a complex life cycle involving organisms at various levels of a trophic web in the marine ecosystem [3]. Fish and squid are the intermediate or paratenic hosts of the parasites, whereas marine mammals mainly cetaceans are the definitive ones [4]. Adult forms for these parasites found in the gastrointestinal tract of a specific vertebrate host and the life cycles of numerous taxa involve the development of larval stages in one or more intermediate hosts [5]. They have a potential risk for human health since these larvae can infect humans after the ingestion of raw or undercooked fish [6–8]. The precise identification of anisakid parasites is essential for their distribution and epidemiology. Berland [9] classified the larvae of anisakid nematodes into two types, namely,* Anisakis* types I and II, based on morphological characteristics such as the length of the ventriculus and presence or absence of mucron at the tip of the tail. Controversy regarding the species names of* Anisakis* type I and II larvae still remains because the larvae lack the detailed morphological characteristics required for precise species identification due to their small size and poor morphological distinctions of these nematodes, particularly their larvae [10, 11]. On the other hand, molecular tools can overcome this limitation and allow the genus- or species-specific identification of anisakids [12]. Recent molecular techniques such as allozyme analysis, polymerase chain reaction-restriction fragment length polymorphism (PCR-RFLP), and sequencing of the internal transcribed spacer (ITS) region (ITS1-5.8S rRNA-ITS2) of ribosomal DNA revealed that the* Anisakis* species consisted of 8 valid species, 5 species:* Anisakis simplex*,* Anisakis pegreffi*,* Anisakis typica*,* Anisakis ziphidarum,* and* Anisakis* sp. and 3 species:* Anisakis physeteris*,* Anisakis brevispiculata,* and* Anisakis paggiae* in the* Anisakis* type I and* Anisakis* type II groupings, respectively [13].

Therefore, the purpose of the present study was to report the occurrence of anisakid larvae in the selected fish specimens from Egyptian water. In addition, identification of the isolated larvae conducted by using morphological studies combined with specific identification using molecular analysis in order to clarify the taxonomic position of the present* Anisakis* species.

## 2. Materials and Methods

### 2.1. Description of the Study Area

Hurghada is one of the most tourist places located on the Red Sea Governorate and far about 399.84 km from Cairo. It stretches for about 36 km along the seashore at 27°15′N, 33°50′E for latitude and longitude, respectively.

### 2.2. Fish Samples Collection and Processing

Sixty specimens of the European hake* Merluccius merluccius lessepsianus* belonged to family Merlucciidae were trapped alive during the period of February to November 2014 from the commercial fishermen from the studied area. The weight and length of the collected samples were ranged between 17–28 kg and 60–110 cm. All specimens were transferred to Laboratory of Parasitology in Faculty of Science at Cairo University, under good aeration. Fish were dissected and then their body cavities and internal organs were examined for the presence of parasitic infections under a stereomicroscope. Parasites were removed, counted, and preserved in 70% ethyl alcohol and then cleared in 5% glycerin for further examination and species identification. For scanning electron microscopy, specimens were fixed at 3% glutaraldehyde, washed in sodium cacodylate buffer, dehydrated in a graded series of ethanol, and then examined and photographed under an Etec Auto scan at 20 KV in Electron Microscope Unit at Faculty of Science, Ain Shams University, Egypt. Measurements were taken with an Olympus ocular micrometer and expressed in millimeter as range followed by mean ± SD in parentheses; otherwise they were stated.

### 2.3. Phylogenetic Analysis

The recovered parasites were removed from ethanol and snap frozen in liquid nitrogen prior to DNA extraction by using QIAamp1 DNA Mini Kit (Qiagen, GmbH, Germany) according to the manufacturer's recommendations. The complete ITS region of approximately 1 kb was amplified by PCR. The reactions were performed with primers NC5 (forward: 5′-TAGGTGAACCTGCGGAAGGATCATT-3′) and NC2 (reverse: 5′-TTAGTTTCTTTTCCTCCGCT-3′) as mentioned by Zhu et al. [20]. PCR products were analyzed by electrophoresis and visualized using UV transilluminator. Selected amplicons were extracted and purified directly using Qiagen Qiaquick (Valencia, CA) columns and then cycle sequenced using ABI BigDye Terminator v3.1 Cycle Sequencing Ready Reaction Kit (Applied Biosystems Inc., Foster City, CA, USA) on automated capillary sequencer (ABI Prism 3100, Applied Biosystems) using the same primers. The sequences were assembled by using DNASTAR module SeqManII. Bootstrap MP Tree was inferred using 1.000 replicates of Branch-and-Bound method.

### 2.4. Ethical Considerations

Animal use followed a protocol approved and authorized by Institutional Animal Care and Use Committee (IACUC) at Zoology Department in Faculty of Science, Cairo University, Egypt.

## 3. Results

Twenty-two (36.66%) out of sixty examined specimens of European hake* M. merluccius lessepsianus* were generally found to be naturally infected with* Anisakis* larvae. The rate of infection was increased during winter to be 60% (18/30) and fall to be 13.33% (4/30) in summer season. These anisakid nematodes were found usually as encapsulated larvae in visceral organs including the peritoneum. The number of larvae per fish ranged from 5 to 30. The mean intensity of the nematode larvae depended on the fish length. The highest intensity was recorded in the hosts with a length >80 cm, which means that the relationship between host length and intensity of infection was positive.

### 3.1. Morphological Description (Figures 1 and 2)

Body of the recovered third-stage larvae was cylindrical in shape, attenuated at both ends, and measured 14.1–25.6 (20.4 ± 1.2) mm in length and 0.48–0.62 (0.55 ± 0.02) mm in width. The larvae covered with a rigid cuticle that has an annular transverse striation that start from the cephalic region and extended to the slit-shaped anus. The lips were inconspicuous, with prominent boring tooth at the anterior extremity. Four small labial papillae (two dorsolateral and two ventrolateral) were surrounding the triradiate mouth opening. Longitudinal lateral grooves are extended along the body and starting next to the mouth area and ended before the mucron. The larvae's oesophagus had anterior muscular part and measured 1.18–2.68 (2.12 ± 0.2) mm long and a glandular ventriculus measured 0.71–0.92 (0.82 ± 0.1) mm long with an oblique esophagointestinal junction. Long intestinal caeca with clear demarcation were present. The excretory duct runs from the excretory pore, which is situated ventrally below the larval tooth. Rectum is surrounded by rectal glands and opens by anal opening. The body of larvae ended at a short mucron which measured 0.019–0.032 (0.025 ± 0.02) mm long.

### 3.2. Molecular Analysis

**Figure 3:**
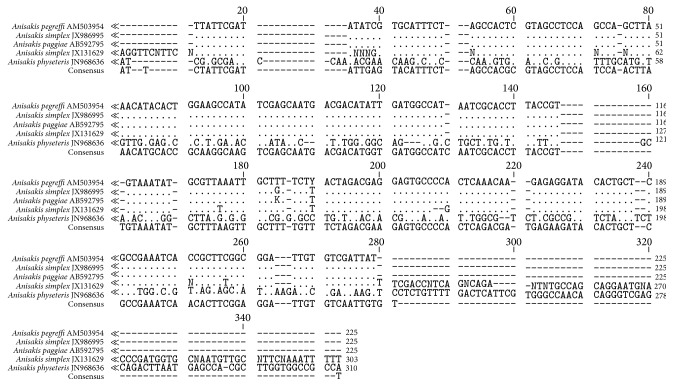
Sequence alignment of* A. simplex* (L3) with most related species. Note: only variable sites are shown. Dots represented bases identical to those of the first sequences and dashes indicate gaps.

**Figure 4:**
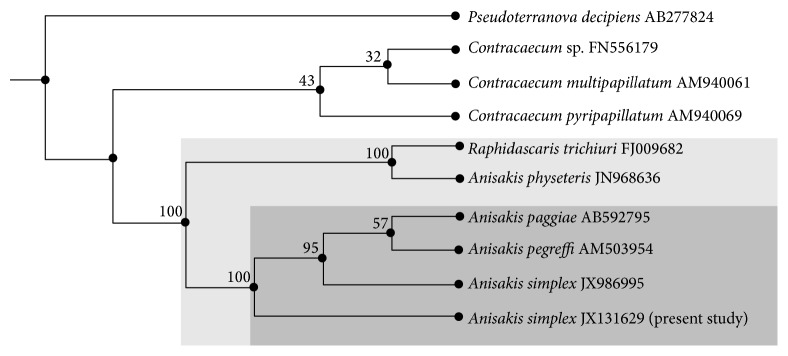
Dendrogram based on SSU rDNA gene sequences showing the phylogenetic relationship between* A. simplex* (L3) and other Anisakidae species. Bootstrap percentages of clades as inferred by ML shown above internal nodes. The SSU rDNA gene sequences of* Pseudoterranova decipiens* were used as an out-group.

A total of 295 bp was obtained with 45.1% GC content for SSU rDNA gene sequences of the present* Anisakis* species. The sequence is deposited in the GenBank under Accession number JX131629. Calculating the percentage of identity between these novel sequences with others retrieved from GenBank demonstrated a high degree of similarity (>84%). This sequence in conjunction with existing data investigates the placement of the present* Anisakis* species within Anisakidae. Comparison of the nucleotide sequences and divergence showed that SSU rDNA of this species revealed the highest blast scores with small number of nucleotide differences with four Anisakidae species, 98% with JX986995* Anisakis simplex* (studied previously), 94% with AM503954* A. pegreffi*, 87% with AB592795* A. paggiae*, and 86% with JN968636* A. physeteris* (Figure 3). The phylogenetic tree is represented by two major clades: the major one clusters all Anisakidae species that were located on a well-supported branch in the ML bootstrap analysis with sequence similarity ranged from 98% to 84% (Figure 4). The minor clade contains AB277824* Pseudoterranova decipiens* as out-group with a high divergence value.

### 3.3. Taxonomic Summary

*Parasite*. The parasite is* Anisakis simplex* third-stage larvae (F: Anisakidae) [21].

*Type Host*. The host is European hake* Merluccius merluccius lessepsianus* Linnaeus [22] (F: Merlucciidae).

*Site of Infection*. The site of infection is visceral organs of infected fish samples.

*Locality*. The location is Hurghada City, Red Sea Governorate, Egypt. 

*Prevalence*. 22 (36.66%) out of 60 specimens of the examined fish were infected. 

*Material Deposition*. Voucher specimens were deposited in Zoology Department, Faculty of Science, Cairo University, Cairo, Egypt.

## 4. Discussion

Anisakis species have complex life cycles which pass through a number of hosts through the course of their lives [23]. Eggs hatch in seawater, and larvae are eaten by crustaceans, usually euphausiids [24, 25]. The infected crustacean is subsequently eaten by a fish or squid, and the nematode burrows into the wall of the gut and encysts in a protective coat, usually on the outside of the visceral organs, but occasionally in the muscle or beneath the skin [20]. The life cycle is completed when an infected fish is eaten by a marine mammal, such as a whale, seal, or dolphin. The nematode excysts in the intestine, feeds, grows, mates, and releases eggs into the seawater in the host's feces. As the gut of a marine mammal is functionally very similar to that of a human, Anisakis species are able to infect humans who eat raw or undercooked fish [26] and they can cause significant clinical diseases [24, 27, 28]. Since the first reports confirming the pathogenic effects of* Anisakis* species in humans [29, 30], there has been increasing awareness of fish-borne parasitic diseases [20, 31, 32]. Anisakidae nematodes have been recorded in the present study with 36.66% as a percentage of infection for the European hake* M. merluccius lessepsianus* represented as high values for larvae abundance; this is coincided with Purivirojkul [33] who stated that larvae of the genus* Anisakis* were found in four fish species (*Muraenesox* sp.,* Epinephelus areolatus*,* Rachycentron canadum*, and* Trichiurus lepturus*) with an infection rate of 52.07%, followed by Pardo-Gandarillas et al. [18] who found a similar prevalence in the European hake from the Atlantic off northwest Africa. Several other studies were shown that anisakid larval stages were mostly found in the musculature of different fish species [34–36], while, in the present study, anisakid larvae penetrate the gut wall and are generally encapsulated on the visceral organs, and peritoneum. The increase in the infection level of* Anisakis* (measured by prevalence, intensity, and abundance) with the age or length of the host reported has often been explained by the accumulation of the parasite throughout the host life and by the increased amount of food ingested by larger fish [37–40]. In the present study this relationship between prevalence and host length/weight can be seen in a positive correlation. The known diversity of the genus has increased greatly over the past 20 years, with the advent of modern genetic techniques in species identification [41]. Each final host species was discovered to have its own biochemically and genetically identifiable “sibling species” of Anisakis, which is reproductively isolated. This finding has allowed the proportion of different sibling species in a fish to be used as an indicator of population identity in fish stocks [42].

Based on morphological characters, nematodes of the present study characterized by the presence of inconspicuous lips possess a prominent boring tooth on the anterior end, and straight gut structure consisting of oesophagus, ventriculus, and intestine, and were identified as* Anisakis* larvae. These results are coincided with data obtained by Rocka [43] in which the third-stage larvae (L3) of* Anisakis simplex* had the above-mentioned characters. The present species were compared morphologically and morphometric with other* Anisakis* species as shown in Table 1. According to the ventriculus length, the presence or absence of mucron can distinguish* Anisakis* type I and type II larvae as mentioned by Setyobudi et al. [17]. Our finding showed that the ventriculus length was 0.71–0.92 (0.82 ± 0.1) mm and the mucron observed and measured 0.019–0.032 (0.025 ± 0.02) mm long; therefore, our measurement data were overlapped with other* A. simplex* described previously by Hurst [14], Larizza and Vovlas [15], Quiazon et al. [16], and Setyobudi et al. [17].

Morphological differentiation of larval and adult stages of anisakid nematodes is difficult, but using molecular identification methods, such as PCR-coupled mutation scanning combined with selective sequencing of the ITS region of the rDNA, has proved to be reliable techniques for the unequivocal identification and differentiation of anisakid nematodes [12, 44, 45]. In the present study, a nuclear rDNA region was amplified by using NC5/NC2 primers mentioned in previous studies by Zhu et al. [20]. ITS-1 and ITS-2 regions representing the* Anisakis* larvae identified herein had the sequence similar to homologous regions within the nuclear ribosomal sequence of* A. simplex* and* A. pegreffi*. Apparently, the tree estimated in this study strongly supported several of the higher taxonomic groups. Anisakidae species represented by the genus* Anisakis* is monophyletic in origin with 100% bootstrap support; this result is in accordance with that obtained by Fagerholm [46] who stated that the rDNA trees (ML and MP), taxa representing the Contracaecinae, Anisakidae, and Raphidascarididae, were each monophyletic and reliably supported by strong bootstrap resampling. On the MP tree, genus-specific clusters with moderate and strong support were delineated in the genera* Anisakis* and* Contracaecum*. The MP tree supported the taxonomic position of the present* Anisakis* species which is deeply embedded in the genus* Anisakis* with a close relationship with* A. simplex* as a more related sister taxon with 100% bootstrap support.

It could be concluded that the parasite species found in* M. merluccius lessepsianus* was* Anisakis* type I larvae and termed as* Anisakis simplex* third-stage larvae with a unique genetic sequence and having new locality records in Egyptian water.

## Figures and Tables

**Figure 1:**
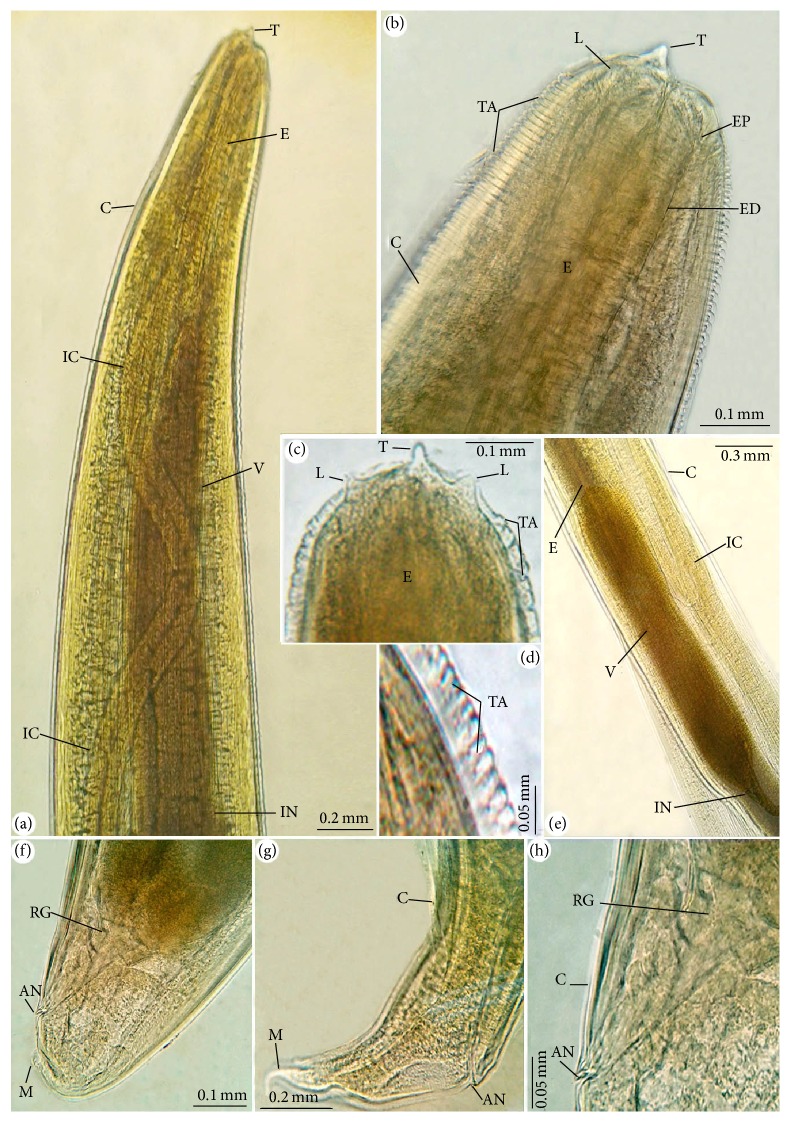
Photomicrographs for different body parts of* Anisakis simplex* (L3). (a)–(c) The anterior end with the boring tooth (T), labia papillae (L), esophagus (E), ventriculus (V), intestine (IN), intestinal caeca (IC), excretory pore (EP), and excretory duct (ED) with high magnifications in (b), (c). (d) The cuticle (C) showing its transverse annulations (TA). (e) Ventriculus (V) with oblique junction with intestine (IN). (f)–(h) Posterior end with anus (AN), rectal gland (RG), and mucron (M) with high magnification in (h).

**Figure 2:**
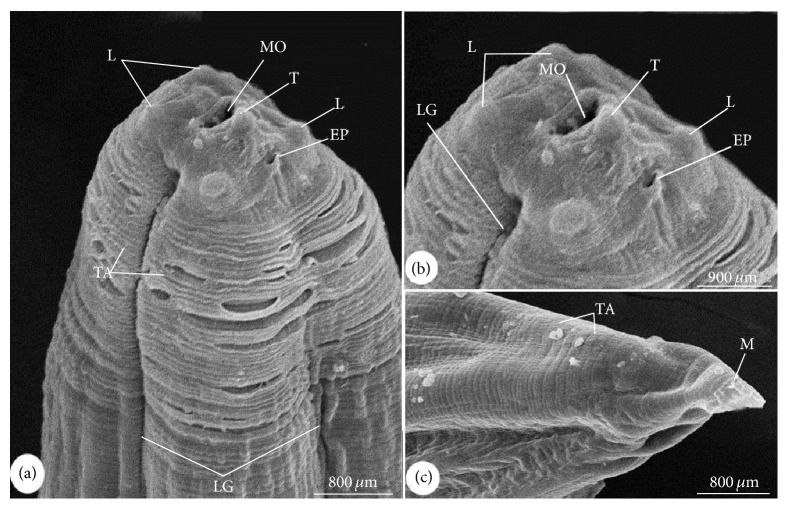
Scanning electron micrographs of* A. simplex* (L3). (a) The anterior part showing a boring tooth (T), labia papillae (L), mouth opening (MO), excretory pore (EP), longitudinal lateral groove (LG), and transverse annulations of cuticle (TA). (b), (c) High magnifications of (b) top view of the anterior part. (c) Posterior extremity with mucron (M).

**Table 1:** Comparative measurements (in millimeters) of the present *A. simplex* (L3) with previously described species.

Related species	Host	Dimensions				
		Body length	Body width	Esophagus length	Ventriculus length	Mucron length
*Anisakis simplex* [14]	*Thyrsites atun*	14–26 (20.26 ± 3.04)	0.29–0.56 (0.43 ± 0.06)	1.57–2.34 (1.99* ± *0.21)	0.47–0.85 (0.69* ± *0.09)	0.015–0.030 (0.023 ± 0.004)
*Anisakis simplex *[15]	*Merluccius merluccius*	15.00–27.50 (21.60 ± 3.47)	0.34–0.51 (0.41 ± 0.05)	2.06–3.08 (2.65 ± 0.29)	0.59–0.92 (0.70 ± 0.08)	0.09–0.14 (0.11 ± 0.01)
*Anisakis pegreffi *[16]	*Delphinus delphis*	11.10–26.78	0.38–0.60	1.04–2.11	0.50–0.78	0.02–0.03
*Anisakis simplex *[16]	*Delphinus delphis*	12.75–29.94	0.45–0.75	1.18–2.58	0.90–1.50	0.02–0.03
*Anisakis simplex *[17]	*Oncorhynchus keta*	19.73–28.41 (23.62 ± 1.87)	0.42–0.68 (0.56 ± 0.04)	1.03–2.59 (2.06 ± 0.25)	0.83–1.56 (1.14 ± 0.13)	0.012–0.031 (0.021 ± 0.004)
*Anisakis physeteris* [18]	*Etmopterus spinax*	27–33	0.63–0.74	1.82–2.89	0.53–0.65	0.17–0.32
*Anisakis paggiae *[19]	*Beryx splendens*	14.00–23.00 (18.22 ± 2.28)	0.41–0.67 (0.54 ± 0.07)	1.24–2.18 (1.59 ± 0.19)	—	—
*Anisakis simplex* (present study)	*M. merluccius lessepsianus*	14.1–25.6 (20.4 ± 1.2)	0.48–0.62 (0.55 ± 0.02)	1.18–2.68 (2.12 ± 0.2)	0.71–0.92 (0.82 ± 0.1)	0.019–0.032 (0.025 ± 0.02)
